# The virome in early life and childhood and development of islet autoimmunity and type 1 diabetes: A systematic review and meta‐analysis of observational studies

**DOI:** 10.1002/rmv.2209

**Published:** 2020-12-30

**Authors:** Clare L. Faulkner, Yi Xuan Luo, Sonia Isaacs, William D. Rawlinson, Maria E. Craig, Ki Wook Kim

**Affiliations:** ^1^ School of Women's and Children's Health University of New South Wales Faculty of Medicine Sydney New South Wales Australia; ^2^ Serology and Virology Division NSW Health Pathology Virology Research Laboratory Prince of Wales Hospital Sydney New South Wales Australia; ^3^ School of Medical Sciences University of New South Wales Sydney New South Wales Australia; ^4^ Faculty of Science School of Biotechnology and Biomolecular Sciences University of New South Wales Sydney New South Wales Australia; ^5^ Institute of Endocrinology and Diabetes Children's Hospital at Westmead Sydney New South Wales Australia; ^6^ Discipline of Child and Adolescent Health University of Sydney Sydney New South Wales Australia

**Keywords:** childhood, islet autoimmunity, next‐generation sequencing, type 1 diabetes, virome

## Abstract

Viruses are postulated as primary candidate triggers of islet autoimmunity (IA) and type 1 diabetes (T1D), based on considerable epidemiological and experimental evidence. Recent studies have investigated the association between all viruses (the ‘virome’) and IA/T1D using metagenomic next‐generation sequencing (mNGS). Current associations between the early life virome and the development of IA/T1D were analysed in a systematic review and meta‐analysis of human observational studies from Medline and EMBASE (published 2000–June 2020), without language restriction. Inclusion criteria were as follows: cohort and case–control studies examining the virome using mNGS in clinical specimens of children ≤18 years who developed IA/T1D. The National Health and Medical Research Council level of evidence scale and Newcastle–Ottawa scale were used for study appraisal. Meta‐analysis for exposure to specific viruses was performed using random‐effects models, and the strength of association was measured using odds ratios (ORs) and 95% confidence intervals (CIs). Eligible studies (one case–control, nine nested case–control) included 1,425 participants (695 cases, 730 controls) and examined IA (*n* = 1,023) or T1D (*n* = 402). Meta‐analysis identified small but significant associations between IA and number of stool samples positive for all enteroviruses (OR 1.14, 95% CI 1.00–1.29, *p* = 0.05; heterogeneity *χ* ^2^ = 1.51, *p* = 0.68, *I* ^2^ = 0%), consecutive positivity for enteroviruses (1.55, 1.09–2.20, *p* = 0.01; *χ* ^2^ = 0.19, *p* = 0.91, *I* ^2^ = 0%) and number of stool samples positive specifically for enterovirus B (1.20, 1.01–1.42, *p* = 0.04; *χ* ^2^ = 0.03, *p* = 0.86, *I* ^2^ = 0%). Virome analyses to date have demonstrated associations between enteroviruses and IA that may be clinically significant. However, larger prospective mNGS studies with more frequent sampling and follow‐up from pregnancy are required to further elucidate associations between early virus exposure and IA/T1D.

**Abbreviations**
50p100K: 50 viral reads per 100,000 raw reads
ADA: American Diabetes Association
CI: confidence interval
CVA: coxsackievirus A
CVB: coxsackievirus B
DIPP: Type 1 Diabetes Prediction and Prevention
DR3: haplotype DRB1*0301‐DQA1*0501‐DQB1*0201
DR4: haplotype DRB1*0401/02/04/05/08‐DQA1*0301‐DQB1*0302/04
DR8: haplotype DRB1*0801‐DQA1*0401‐DQB1*0402
DR3/4: heterozygous genotype comprising both DR3 and DR4 haplotypes
ECHO: enteric cytopathic human orphan virus
ENDIA: Environmental Determinants of Islet Autoimmunity
EV: enterovirus
EV‐A: enterovirus A
EV‐B: enterovirus B
FDR: first‐degree relative
GADA: – glutamic acid decarboxylase 65 autoantibodies
HLA: human leukocyte antigen
IA: islet autoimmunity
IAA: insulin autoantibodies
IA2A: tyrosine phosphatase‐like insulinoma antigen 2 autoantibodies
ICA: islet cell autoantibodies
mNGS: metagenomic next‐generation sequencing
NHMRC: National Health and Medical Research Council
NOS: Newcastle–Ottawa quality assessment scale
OR: odds ratio
PBMC: peripheral blood mononuclear cell
PRISMA: Preferred Reporting Items for Systematic Reviews and Meta‐Analyses
T1D: type 1 diabetes mellitus
TEDDY: The Environmental Determinants of Diabetes in the Young
VirCapSeq‐VERT: Virome Capture Sequencing Platform for Vertebrate Viruses
*χ*2: Cochrane's *Q* test
ZnT8A: zinc transporter 8 autoantibodies

## 1 INTRODUCTION

Type 1 diabetes (T1D) is common, affecting more than 600,000 children aged <15 years worldwide.^1^ T1D is preceded by islet autoimmunity (IA) lasting months to decades.^2^ It is defined serologically as multiple autoantibodies against one or more T1D‐associated autoantigens, including insulin (IAA), glutamic‐acid decarboxylase (GADA), tyrosine phosphatase‐like insulinoma antigen 2 (IA2A), islet cell cytoplasmic proteins (ICA) and β‐cell‐specific zinc transporter 8 (ZnT8A).^3^ T1D pathogenesis results from a complex interplay of genetic predisposition^4^ ^,^ ^5^ and environmental exposures.^6^ ^,^ ^7^ Accumulating evidence supports the influence of environmental factors, particularly viruses. The increased incidence of T1D is too high to be attributed to genetics alone,^1^ ^,^ ^8^ with data showing seasonal IA/T1D clustering,^9^ geographical variation in incidence^10^ and more frequent in utero and early‐life infections in affected individuals.^11^ ^,^ ^12^

Higher rates of enterovirus (EV) infection, detected by serological^13^ ^,^ ^14^ or molecular methods,^15^, ^16^, ^17^ have been observed in T1D patients at diagnosis versus unaffected controls, or prospectively in individuals who subsequently develop IA and/or T1D versus those who do not. Accordingly, our previous meta‐analysis investigating EV using molecular methods demonstrated significant association between EV and IA (odds ratio [OR] 3.7, 95% confidence interval [CI] 2.1–6.8, *p* < 0.001) or T1D (9.8, 5.5–17.4, *p* < 0.001).^18^ In addition, EV proteins and RNA have been isolated from pancreata of affected patients, with upregulated EV receptors selectively expressed in pancreatic islets.^19^ ^,^ ^20^ However, inconsistencies in findings^21^, ^22^, ^23^ make it difficult to establish a definitive causal association. Importantly, substantial investigation bias exists for EVs in previous studies.^22^ In contrast, only a limited number of studies have reported on the potential associations of other viruses with T1D, including mumps,^24^ cytomegalovirus,^25^ rotavirus,^26^ parechovirus,^27^ ^,^ ^28^ Epstein–Barr virus,^29^ rubella^12^ and parvovirus.^30^

In an effort to alleviate this bias towards EVs, a growing number of studies are applying high‐throughput metagenomic next‐generation sequencing (mNGS) to comprehensively characterise the population of all known human viruses (the ‘virome’), simultaneously. Here, we report the first systematic review and meta‐analysis of observational studies using mNGS to investigate vertebrate‐infecting DNA and RNA viruses in children ≤18 years, and subsequent development of IA or T1D. Analysis of bacteriophage has been excluded from this review. The unbiased viral mNGS in early life and childhood has potential to comprehensively identify diabetogenic viruses increasing the IA/T1D risk or viruses affording protection. This may present new opportunities to intervene through antiviral medications or vaccination.

## 2 METHODS

### 2.1 Search strategy and selection criteria

This review is registered on PROSPERO (23 July 2020), registration number CRD42020188737. Two reviewers (Clare L. Faulkner and Yi Xuan Luo) independently conducted a systematic search for observational studies investigating the association between virome composition and/or abundance, and IA or T1D. EMBASE and MEDLINE databases were searched (2000–1 June 2020) using the strategy in Appendix S1. The search was performed without geographical or language restrictions and limited to studies in humans. Restriction to studies published from year 2000 onwards was informed by emergence of mNGS and other viral sequencing technologies.^31^ ^,^ ^32^ This search was supplemented by manual searching of references of identified papers, key journals, OpenGrey and ProQuest to identify additional articles potentially missed by online indexes. PROSPERO was interrogated to confirm no recent/ongoing systematic reviews.

Eligible studies were observational (cohort, case–control and nested case–control; including letters or abstracts), using mNGS to characterise the virome in any clinical specimen in children aged ≤18 years who developed IA and/or T1D. Age restriction was imposed because IA often develops in childhood, suggesting viruses exert influence early in life. IA was defined as persistence of one or more autoantibodies against T1D‐associated autoantigens (IAA, GADA, IA2A, ICA and ZnT8A) in ≥2 time‐separated consecutive samples. Transplacental autoantibodies were excluded, defined as transient presence of the same autoantibody in a child <18 months and his/her mother. T1D was defined using American Diabetes Association criteria.^33^ Eligible studies were categorised into two groups based on the outcome: IA or T1D. Data were extracted on vertebrate‐infecting viruses only, excluding studies that only analysed bacteriophage.

**FIGURE 1:**
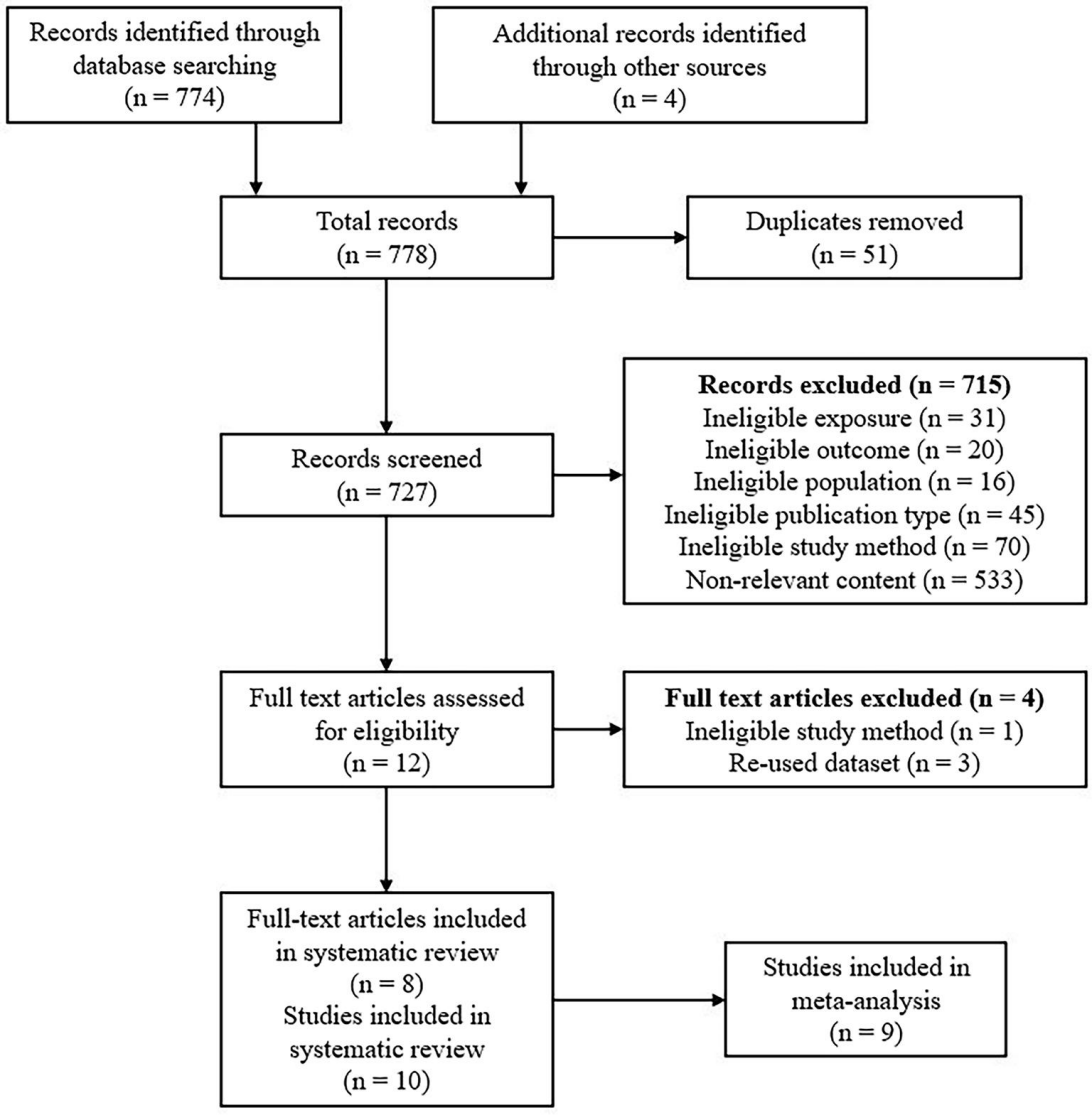
Flow diagram of study selection

Two reviewers (Clare L. Faulkner and Yi Xuan Luo) screened titles and abstracts of identified studies (Figure 1) and then analysed shortlisted studies in full text for eligibility. In instances of uncertainty (*n* = 2), an independent advisor (Ki Wook Kim) was consulted to reach consensus decision. Case reports/series, uncontrolled studies, reviews and animal studies were omitted based on exclusion criteria.

### 2.2 Data analysis

Data extracted included publication authors, year and geographical location; study design; study participants; number of cases/controls; age; level of pre‐existing IA/T1D risk (human leukocyte antigen [HLA] genotype and family history); sample type, number and collection protocols; virus detection method and positivity threshold; rates of virus positivity in cases/controls; examined outcome (IA or T1D); measures of effect and funding. Original authors were contacted for insufficient or missing published data (*n* = 4).

Two reviewers (Clare L. Faulkner and Yi Xuan Luo) independently assessed the quality of included studies using the National Health and Medical Research Council (NHMRC) level of evidence scale^34^ and the Newcastle–Ottawa Quality Assessment Scale (NOS),^35^ as recommended by Cochrane collaboration. The NHMRC scale grades the study design according to a defined research hierarchy. The NOS evaluates three areas: selection, comparability and exposure; out of nine points, greater than six indicates good methods. Our chosen comparability controls were age and sampling time, and two critical factors likely to impact the prevalence of viruses.

We calculated ORs with 95% CIs and *p‐*values for viruses present in children with IA or T1D versus controls from the extracted data using the Mantel–Haenszel method. Virus positivity was defined as virus material present in ≥1 study sample as detected by mNGS. Analysis was performed both for the number of case/control individuals and the number of case/control samples positive for virus. We used both fixed‐ and random‐effects models; only results from random‐effects models are presented due to heterogeneity of study populations. Statistical heterogeneity was explored using Cochrane's *Q* Test (*χ* ^2^) and the *I* ^2^ statistic, which indicate the proportion of variance of the summary effect attributable to between‐study heterogeneity. A *p* < 0.10 was considered a statistically significant heterogeneity, while *I* ^2^ ≤ 25% and ≥75% were deemed low and high heterogeneity, respectively. Subgroup analyses were performed for geographical location, stool versus plasma, consecutive virus shedding and studies using comparable detection thresholds, and pooled ORs were calculated. Sensitivity and influence analyses were conducted by the study size. Data analysis was completed in Review Manager, Version 5.4 (Cochrane Collaboration),^36^ with significance *p* ≤ 0.05.

Our systematic review is reported using meta‐analysis of observational studies in epidemiology and Preferred Reporting Items for Systematic Reviews and Meta‐Analyses guidelines.

## 3 RESULTS

The search returned 778 publications (51 duplicates), leaving 727 articles for review. Title and abstract screening identified 12 publications for full‐text review. Four were excluded: three were repeat data sets^37^, ^38^, ^39^; one used targeted polymerase chain reaction (PCR) rather than pre‐specified mNGS.^40^ Eight were included—one abstract,^41^ two letters^42^ ^,^ ^43^ and five articles^44^, ^45^, ^46^, ^47^, ^48^ (Figure 1). Two publications^42^ ^,^ ^47^ contained two study groups that were analysed separately, giving a total 10 studies with 1,425 participants (695 cases, 730 controls). Nine were nested case–control studies using samples collected within prospective birth cohorts, eight investigated IA (510 cases, 513 controls) and two investigated T1D (185 cases, 217 controls; Tables 1 and 2). One IA study^48^ with insufficient data was excluded from meta‐analysis.

### 3.1 Study characteristics

Six studies defined IA as positivity for ≥1 T1D‐associated autoantibody; two defined as ≥2 autoantibodies (Table 2). All IA studies, except two,^43^ ^,^ ^46^ required persistent autoantibody positivity across consecutive visits. All IA and T1D nested case–control studies selected participants from within the same prospective cohort. Most prospective cohorts recruited participants with high‐risk HLA genotypes (DR3/4, DR4/4, DR4/8 and DR3/3), except one^47^ that recruited children with ≥1 first‐degree relative (FDR) with T1D, and one^43^ that required both the criteria. Most studies analysed children less than 6 years; two investigated older children ≤10^42^ and ≤18^41^ years. Most studies included <50 participants.^43^, ^44^, ^45^, ^46^, ^47^, ^48^ Study characteristics are summarised in Tables 1 and 2.

Seven studies examined the gut virome by sequencing virus‐enriched stool^41^ ^,^ ^42^ ^,^ ^44^ ^,^ ^46^, ^47^, ^48^; two investigated plasma^45^ and one examined peripheral blood mononuclear cells (PBMCs).^43^ Alongside mNGS, four studies utilised specific PCR for common viruses.^41^ ^,^ ^44^ ^,^ ^47^ Three studies employed Virome Capture Sequencing Platform for Vertebrate Viruses (VirCapSeq‐VERT) to enhance sensitivity for vertebrate‐infecting viruses.^43^ ^,^ ^47^ Two studies cultivated stool in virus‐susceptible cells to amplify low‐abundance EV or other common viruses.^42^

Viruses commonly reported in the IA group included EV, bocaparvovirus, anelloviruses, parechovirus, rotavirus, sapovirus, cardiovirus and mastadenovirus. Norovirus, circovirus, mamastrovirus, kobuvirus, picobirnavirus, erythroparvovirus and roseolovirus positivity were only reported in two studies, and eight other viruses were reported each in only one study. Viruses commonly reported in the two T1D studies were EV, parechovirus, bocaparvovirus, anelloviruses, sapovirus, cardiovirus, mastadenovirus, norovirus and mamastrovirus. Kobuvirus and circovirus were only reported in one study.^41^

### 3.2 Quality of evidence

The NOS scores were ≥8 (Table 3), indicating good methodological quality overall. Of the 10 studies, only 1 IA and 1 T1D study^42^ adjusted for potential confounders, including HLA genotype, lifestyle factors, demographic factors and factors related to the child.

**TABLE 1:** Summary of studies investigating the virome and IA

Study	Country	Cases	Cases/controls	Autoantibodies measured	Age (years)^a^	Control matching	Sample type	Sample collection protocol	Total samples (cases/controls)	Virus sequencing; detection threshold
Cinek et al.^48^	Finland	Children who seroconverted less than 2 years old from DIPP	18/18	≥2 IAA, GADA, ICA, IA2A, or ZnT8A	0–2.5	Date/place of birth, HLA genotype, gender	Stool	3, 6 and 9 months before IA onset	92 (46/46)	mNGS only; threshold 50p100K
Hippich et al.^43^	Germany	Ab+ children from BABYDIET	20/20	≥1 IAA, GADA, IA2A or ZnT8A	0–1.2	Age	PBMCs	3 Monthly from age 3 months	102 (51/51)	VirCapSeq‐VERT and mNGS; threshold not stated
Kim et al.^47^	Australia	Ab+ children from VIGR	20/20 (Stool study)	≥1 IAA, GADA or IA2A	5.7 ± 3.7	Age, gender	Stool	At seroconversion and/or within 15 ± 6 months prior	64 (32/32)	VirCapSeq‐VERT and mNGS; two thresholds: (1) 100 viral reads matched at species level and (2) 50p100K
			41/41 (Plasma study)				Plasma	At seroconversion and/or within 13 ± 4 months prior	118 (59/59)	
Kramná et al.^44^	Finland	Children who seroconverted less than 2 years old from DIPP	19/19	≥2 IAA, GADA, ICA, IA2A or ZnT8A	0–2	Date/place of birth, HLA genotype, gender	Stool	3, 6 and 9 months before IA onset	96 (48/48)	mNGS and retesting with PCR; threshold 50p100K
Vehik et al.^42^	United States, Finland, Germany, Sweden	Ab+ children from TEDDY	383/383	≥1 IAA, GADA or IA2A	0–10	Age, clinical centre, gender, T1D family history	Stool	Monthly from age 3–48 months, quarterly thereafter; mean samples per subject 9	8,654 (4,327/4,327)	Culture to amplify low abundance viruses and mNGS; VirMAP aggregate bit score of 400 as threshold
Zhao et al.^46^	Finland and Estonia	Ab+ children from DIABIMMUNE	11/11	≥1 IAA, GADA, IA2A, ICA or ZnT8	0–3	Age, gender, HLA genotype, birth delivery method, country	Stool	Monthly from 0 to 3 years; sequential samples analysed	220 (114/106)	mNGS; threshold not stated
Lee et al.^45^	USA, Finland, Germany, Sweden	Ab+ children with rapid‐onset T1D from TEDDY	14/14	≥1 IAA, GADA or IA2A	0–3	Age, clinical centre, T1D family history	Plasma	Last Ab− negative visit and first Ab+ seroconversion visit	56 (28/28)	mNGS; threshold not stated

Abbreviations: 50p100K, 50 viral reads per 100,000 raw reads; Ab−, autoantibody negative; Ab+, autoantibody positive; DIPP, Type 1 Diabetes Prediction and Prevention; FDR, first‐degree relative; GADA, glutamic‐acid decarboxylase autoantibodies; HLA, human leukocyte antigen; IA, islet autoimmunity; IA2A, tyrosine phosphatase‐like insulinoma antigen 2 autoantibodies; IAA, islet autoantibodies; ICA, islet cell autoantibodies; mNGS, metagenomic next‐generation sequencing; PBMC, peripheral blood mononuclear cell; T1D, type 1 diabetes; TEDDY, The Environmental Determinants of Diabetes in the Young; VIGR, Australian Viruses in the Genetically at Risk; VirCapSeq‐VERT, Virome Capture Sequencing Platform for Vertebrate Viruses; ZnT8A, β‐cell‐specific zinc transporter 8 autoantibodies.

^a^ Data are reported as range, or mean ± SD.

**TABLE 2:** Summary of studies investigating the virome and T1D

Study	Country	Cases/controls	Design/eligibility	Age (years)	Controls	Sample collection protocol	Total samples (cases/controls)	Virus sequencing and detection threshold
Cinek et al.^41^	Azerbaijan, Jordan, Nigeria, Sudan	73/105	Case control; patients with newly diagnosed T1D	<18	Matched for age, place of residence	One stool sample collected shortly after T1D diagnosis	177 (73/104)	mNGS and specific PCR for EV, parechovirus, adenovirus, bocavirus, norovirus, sapovirus; threshold not stated
Vehik et al.^42^	USA, Finland, Germany, Sweden	112/112	Nested‐case control; high‐risk HLA genotypes^a^	0–10	Matched for age, clinical centre, gender, T1D family history	Stool samples collected monthly from age 3 to 48 months, quarterly thereafter	3,380 (1,690/1,690)	Culture to amplify low abundance viruses and mNGS; VirMAP aggregate bit score of 400 as threshold

Abbreviations: EV, enterovirus; HLA, human leukocyte antigen; mNGS, metagenomic next‐generation sequencing; PCR, polymerase chain reaction; T1D, type 1 diabetes.

^a^ High‐risk HLA genotypes include DR3/4, DR4/4, DR4/8 and DR3/3.

**TABLE 3:** Quality of evidence in observational studies investigating the virome and islet autoimmunity or type 1 diabetes

Study	NHMRC level of evidence^a^	Newcastle–Ottawa Scale Score				Cases and controls matched?					Details of virome sequencing Method given?
		Selection	Comparability	Exposure	Total/Nine	Age	Sex	HLA	Place	Sample time	
Cinek et al.^48^	II	●●●●	●●	●●●	9	Yes	Yes	Yes	Yes	Yes	Yes
Cinek et al.^41^	III‐3	○●●●	●●	●●●	8	Yes	No	No	Yes	Yes	Yes^b^
Hippich et al.^43^	II	●●●●	●○	●●●	8	Yes	N/A	N/A	N/A	N/A	Yes
Kim et al.^47^	II	●●●●	●●	●●●	9	Yes	Yes	No	Yes	Yes	Yes
Kramná et al.^44^	II	●●●●	●●	●●●	9	Yes	Yes	Yes	Yes	Yes	Yes
Lee et al.^45^	II	●●●●	●●	●●●	9	Yes	No	No	Yes	Yes	Yes
Vehik et al.^42^	II	●●●●	●●	●●●	9	Yes	Yes	Yes	Yes	Yes	Yes
Zhao et al.^46^	II	●●●●	●●	●●●	9	Yes	Yes	Yes	Yes	Yes	Yes

*Note*: ● = 1 point; N/A, not available.

^a^ II, nested case‐control study; III‐3, case‐control study.

^b^ Not referenced.

### 3.3 Islet autoimmunity

**FIGURE 2:**
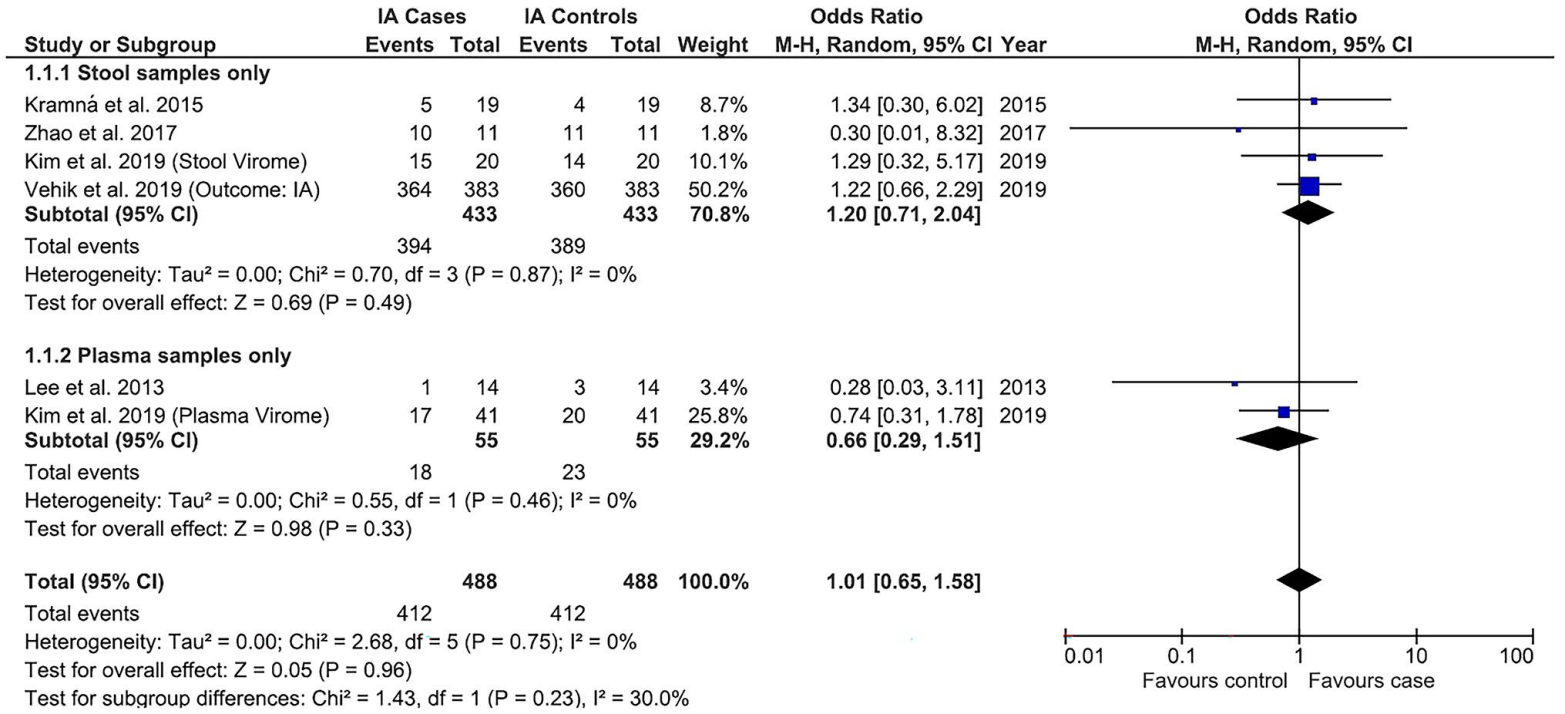
Individual and summary odds ratios for positivity for any vertebrate‐infecting virus in children with islet autoimmunity (IA) versus no IA, with stool versus plasma subgroup analysis. All results based on rates of virus positivity as detected by metagenomic next‐generation sequencing. No associations were found between virus positivity in stool or plasma and childhood IA

Seven studies investigated vertebrate‐infecting viruses and IA. No significant heterogeneity was observed, unless stated. Due to insufficient sample data, positivity for any virus was only analysed at the individual level, with no difference between cases and controls, pooled OR 1.03 (95% CI 0.67–1.60, *p* = 0.89). Stool versus plasma subgroup analysis gave pooled ORs 1.20 (0.71–2.04, *p* = 0.49) and 0.66 (0.29–1.51, *p* = 0.33), respectively (Figure 2); the PBMC study^43^ was excluded. For European versus non‐European (Australian) subgroup analyses, pooled ORs were 1.14 (0.66–1.96, *p* = 0.65) and 0.87 (0.41–1.82, *p* = 0.71), respectively.

**FIGURE 3:**
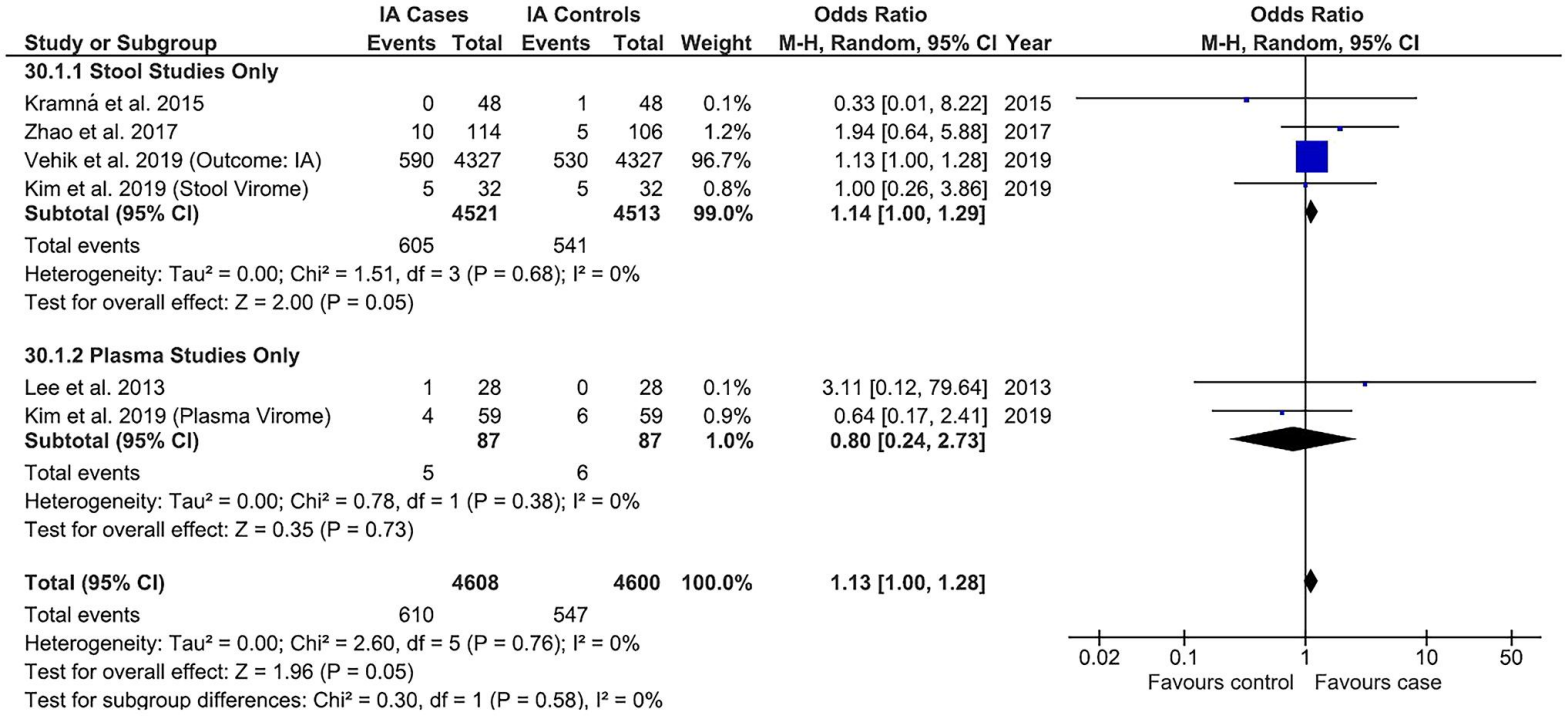
Individual and summary odds ratios for number of samples positive for enterovirus (EV) in children with islet autoimmunity (IA) versus no IA, with stool versus plasma subgroup analysis. All results based on rates of virus positivity as detected by metagenomic next‐generation sequencing. An association was found between childhood IA and number of stool samples positive for EV (odds ratio 1.14; 95% confidence interval 1.00–1.29, *p* = 0.05; heterogeneity *χ* ^2^ = 0.50, *p* = 0.68, *I* ^2^ = 0%), but not the number of plasma samples positive for EV

Meta‐analyses for specific viruses were conducted where proportions of positive case–control individuals or samples were reported in ≥1 study. Six studies found a significant association between the number of EV‐positive samples and IA (1.13, 1.00–1.28, *p* = 0.05). For stool versus plasma subgroup analysis, the pooled ORs were 1.14 (1.00–1.29, *p* = 0.05) and 0.80 (0.24–2.73, *p* = 0.73), respectively (Figure 3). There was no association between EV‐positive individuals and IA (1.13, 0.86–1.48, *p* = 0.37). For stool versus plasma subgroup analysis, pooled ORs were 1.15 (0.87–1.51, *p* = 0.32) and 0.80 (0.23–2.77, *p* = 0.72), respectively. There were minimal differences in effect sizes with sensitivity analysis.

**FIGURE 4:**
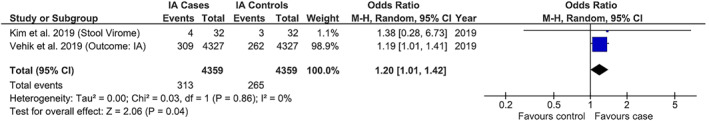
Individual and summary odds ratios (ORs) for number of stool samples positive for enterovirus B (EV‐B) in children with islet autoimmunity (IA) versus no IA. All results based on rates of virus positivity as detected by metagenomic next‐generation sequencing. An association was found between number of stool samples positive for EV‐B and IA (OR, 1.20; 95% confidence interval 1.01–1.42, *p* = 0.04; heterogeneity *χ* ^2^ = 0.03, *p* = 0.86, *I* ^2^ = 0%)

Two studies^42^ ^,^ ^47^ investigated EV subtypes, EV‐A and EV‐B, in stool, with significant association between IA and number of EV‐B‐positive samples (1.20, 1.01–1.42, *p* = 0.04; Figure 4), but not individuals (0.99, 0.74–1.32, *p* = 0.94). There were no associations between IA and EV‐A‐positive samples or individuals, pooled ORs 1.61 (0.43–5.94, *p* = 0.48; significant heterogeneity *χ* ^2^ = 2.81, *p* = 0.09, *I* ^2^ = 64%) and 1.12 (0.84–1.50, *p* = 0.42), respectively.

**FIGURE 5:**
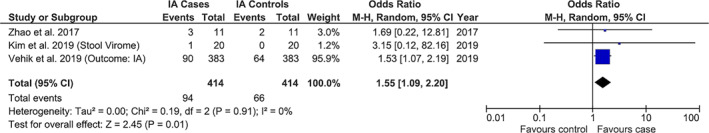
Individual and summary odds ratios (ORs) for studies examining positivity for enterovirus (EV) in ≥2 consecutive stool samples in children with islet autoimmunity (IA) versus no IA. All results based on rates of virus positivity as detected by metagenomic next‐generation sequencing. An association was found between consecutive EV shedding and childhood IA (OR 1.55, 95% confidence interval 1.09–2.20, *p* = 0.01; heterogeneity *χ* ^2^ = 0.10, *p* = 0.91, *I* ^2^ = 0%)

Three stool studies^42^ ^,^ ^46^ ^,^ ^47^ found significant association between consecutive EV shedding (≥2 sequential samples positive) and IA, pooled OR 1.55 (1.09–2.20, *p* = 0.01; Figure 5). One study^42^ reported consecutive EV‐A/EV‐B shedding: IA was associated with consecutive EV‐B (2.46, 1.46–4.16, *p* = 0.0007), but not EV‐A (1.19, 0.74–1.92, *p* = 0.47).

Four studies reported parechovirus positivity in stool. There was no association between the number of individuals positive for parechovirus and IA, pooled OR 0.83 (0.63–1.10, *p* = 0.20). However, for parechovirus‐positive samples and IA (0.66, 0.32–1.35; *p* = 0.25), there was significant heterogeneity (*χ* ^2^ = 3.88; *p* = 0.009; *I* ^2^ = 74%). In influence analysis, the removal of the largest study outlier (>8,000 samples) strengthened the magnitude of association, pooled OR 0.44 (0.23–0.81, *p* = 0.008) and low heterogeneity (*χ* ^2^ = 1.10, *p* = 0.33, *I* ^2^ = 9%). There was minimal difference in effect size for the number of individuals positive for parechovirus.

Meta‐analyses at the individual and sample level for rotavirus, bocaparvovirus, anelloviruses, sapovirus, norovirus, cardiovirus, circovirus, mamastrovirus, mastadenovirus, kobuvirus, picobirnavirus, erythroparvovirus and roseolovirus showed no associations with IA (Table S1). There were minimal differences in effect sizes with sensitivity and influence analyses. Viruses reported in only one study were precluded from the meta‐analysis. One study^42^ conducted strain‐specific analysis for mastadenovirus (human mastadenovirus A, C, F) and found association between the number of samples with positive human mastadenovirus F and IA, OR 1.33 (1.08–1.54, *p* = 0.007).

Meta‐analysis of consecutive shedding of parechovirus, bocaparvovirus, anelloviruses and picobirnavirus found no associations with IA (Table S2). Meta‐analysis of viruses reported in the two studies^44^ ^,^ ^47^ applying the same positivity threshold of 50 viral reads per 100,000 raw reads (50p100K) found no associations between any virus and IA, including for EV, parechovirus, anelloviruses, bocaparvovirus and sapovirus (Table S3).

Study quality subgroup analysis was excluded as NOS scores were ≥8. HLA subgroup analysis was excluded as no studies stratified virus positivity by genotype, and all but two^47^ recruited only high‐risk genotypes. Geographical location subgroup analysis for individual viruses was excluded as only two studies were non‐European^47^ and multicentre studies insufficiently compared study populations.

Only one study^47^ examined differential abundance of viruses in the gut of children with IA versus controls, precluding meta‐analysis. It found 129 viruses with more than twofold difference in cases versus controls (*p* = 0.02). Notably, human mastadenovirus F, astrovirus, human adenovirus 41, coxsackievirus A2 (CVA2), enteric cytopathic human orphan virus 30, coxsackievirus B3 and human parechovirus were more abundant in cases, while saffold virus, norovirus and rotavirus A were more abundant in controls. Every differentially abundant EV‐A (CVA2, 5, 6, 8, 14) was more abundant in cases (*p* < 0.00001). Additionally, one study^46^ measured intestinal viral alpha and beta diversity in children with IA versus controls, with the gut viromes of cases significantly less diverse (with lower interpersonal variation) compared to controls (*p* < 0.001).

One analysis of very young children <6 months^42^ demonstrated the association between early‐life human mastadenovirus C infection and lower IA risk (0.55, 0.38–0.80, *p* = 0.001).

### 3.4 Type 1 diabetes

Two studies^41^ ^,^ ^42^ investigated gut vertebrate‐infecting viruses and T1D, with no association between positivity for any virus and T1D, pooled OR 0.94 (0.54–1.64, *p* = 0.83; and no heterogeneity *χ* ^2^ = 0.21, *p* = 0.65, *I* ^2^ = 0%).

Meta‐analyses for EV, parechovirus, cardiovirus, norovirus, sapovirus, mastadenovirus, bocaparvovirus, mamastrovirus and anelloviruses were not significant for the number of positive individuals (Table S4). Strain‐specific analysis for mastadenovirus produced no significant effect sizes, including for human mastadenovirus A (OR 0.92, 0.51–1.65, *p* = 0.78) and human mastadenovirus F (OR 0.72, 0.42–1.24, *p* = 0.24), with no heterogeneity between studies. One study^42^ reported virus‐positive sample numbers, precluding meta‐analysis. One study^42^ analysed EV subtypes, reporting an association between T1D protection and number of EV‐B positive samples (0.73, 0.53–0.99, *p* = 0.05), but not individuals (0.69, 0.41–1.18, *p* = 0.18); EV‐A was not associated with T1D. Limited studies precluded subgroup and sensitivity analyses.

## 4 DISCUSSION

This systematic review of 10 observational studies, involving 695 cases and 730 controls, demonstrated associations between virome composition in children ≤18 years and development of IA, but not T1D. This suggests early virome changes may influence initiation of IA, but not progression to T1D. There was a weak association between the number of stool samples positive for EV or EV‐B and IA, with approximately 1.2 times the odds of EV or EV‐B positivity in children who developed IA versus controls; ORs 1.14 (1.00–1.29) and 1.20 (1.01–1.42), respectively. There was 1.5 times the odds of consecutive EV shedding in stool of children with IA versus controls; OR 1.55 (1.09–2.20). Only one study measured consecutive shedding of EV serotypes,^42^ demonstrating significant association between consecutive EV‐B positivity and IA (2.46; 1.46–4.16), but not EV‐A (1.19, 0.74–1.92). Influence analysis, removing the largest outlier study, demonstrated half the odds of parechovirus shedding in stool for children with IA versus controls (0.44, 0.23–0.81). Other viruses were not associated with IA at the individual or sample level.

These data suggest that specific gut vertebrate‐infecting viruses present in the gut virome, rather than the presence of any virus, influence IA risk. Research repeatedly reports associations between EV infection and IA initiation, supported by EV RNA in stools^40^ and EV RNA/antibodies in sera.^16^ ^,^ ^40^ ^,^ ^49^ ^,^ ^50^ Our results support clinical studies^51^ ^,^ ^52^ and pancreatic tropism studies^20^ ^,^ ^53^ ^,^ ^54^ favouring EV‐B as a candidate virus in IA susceptibility. In contrast, studies of other candidate viruses remain inconclusive. For example, parechovirus may confer IA protection; however, no associations with IA/T1D have previously been reported.^27^ ^,^ ^28^

We demonstrated an association between IA and the number of virus‐positive samples, but not individuals. Thus, when an individual has more than one positive sample, their IA risk is amplified. This may relate to viral persistence, protracted infection or increased exposure through reinfection. In particular, IA was associated with consecutive or prolonged EV/EV‐B shedding. Consecutive shedding is a strong indicator of persistent infection. Viral persistence is suggested to play a critical role in the development of autoimmunity^22^ ^,^ ^55^ ^,^ ^56^ through ongoing aberrant presentation of antigens to the immune system, production of inflammatory cytokines and induction of Endoplasmic Reticulum stress.^57^ ^,^ ^58^ The gut mucosa may be a potential viral reservoir for sustained pancreatic infection^59^ with multiple virus‐positive stool samples a marker of persistent gut infection. Consecutive shedding may also indicate defective or dysregulated innate immune defence, which increases autoimmune propensity. Longitudinal virome studies are therefore essential in tracking virus infections over time. However, our ability in this review to distinguish between persistence of the same viral strain or reinfection in consecutively positive patient samples was limited by most studies reporting viruses detected at the genus level and intermittent sampling across studies.

The one study that conducted differential abundance analysis found 129 viruses with a ≥2‐fold difference in abundance in the gut of IA cases versus controls.^47^ This suggests IA risk is closely linked to viral load of a variety of viruses.^58^ ^,^ ^60^ Higher viral titre facilitates greater replication, pancreatic transmission, persistence, cellular stress and establishment of an immunogenic environment.^47^ ^,^ ^61^ ^,^ ^62^ Future mNGS studies in larger cohorts with more timepoints preceding IA or T1D are required to elucidate IA/T1D‐associated vertebrate‐infecting viruses and compare differential abundance across the breadth of potentially diabetogenic viruses. Additionally, further research investigating virome composition across defined early‐life stages is required to determine time points where viruses exert greatest influence.^9^ ^,^ ^63^ Only one study^46^ measured ‘virome diversity’, finding lower diversity in children with IA. Further development and standardisation of these diversity measures are required to facilitate comparability between studies and greater understanding of association between virome composition and IA/T1D risk.

### 4.1 Strengths and weaknesses

To minimise bias, we implemented pre‐defined eligibility criteria, screening by independent reviewers, no language restrictions and sources beyond indexed databases. Random‐effects models may have provided more conservative effect estimates by accounting for study population heterogeneity and generating wider confidence intervals.^64^ ^,^ ^65^ Limiting investigation to children ≤18 years may have skewed results due to high rates of childhood background infection. However, there are no adult IA/T1D virome studies. We included studies conducted globally to minimise geographical bias related to infection rates. However, eight studies were European, where T1D incidence is the highest.^1^ This precluded country subgroup analysis in most meta‐analyses. All studies recruited infants with high‐risk HLA or an affected FDR, potentially reducing generalisability.

Our findings have limitations. Only two studies examined T1D, limiting the analysis of associations between viruses and progression to T1D beyond IA initiation. Of the two studies that analysed T1D, one analysed prospectively collected samples and one analysed samples collected at/after T1D diagnosis. This may limit comparability due to potential differences when examining the virome after, rather than before, diagnosis of the study outcome. IA was predominantly defined as ≥1 autoantibody, despite single autoantibody conferring lower lifetime T1D risk versus multiple antibodies.^66^ ^,^ ^67^ However, our stratification of results by autoantibody number was precluded by insufficient data. Most studies matched for HLA genotype, but HLA subgroup analysis could not be conducted, despite HLA predicting IA/T1D risk^4^ ^,^ ^68^ and potentially influencing virus‐induced pathology, immune dysregulation or susceptibility to viral infection.^60^ However, studies that have explored the association with HLA and EV infection specifically have reported inconsistent results, finding varying infection prevalence in individuals with different HLA genotypes^69^ and no association.^70^ Thus, future studies of infants with a range of low‐ to high‐risk genotypes are required, such as Environmental Determinants of Islet Autoimmunity (ENDIA). Many studies did not account for other potential environmental risk factors, such as anthropometry,^71^ diet,^72^ vitamin D,^73^ omega‐3 fatty acids,^74^ birth delivery route^75^ and/or breastfeeding,^76^ all of which can influence the viral presence and IA/T1D risk. However, controlling for all potential confounders in small case–control studies remains challenging, with only the two largest studies^42^ included in this review reporting adjustment for a number of potential confounders.

Studies applied different positivity thresholds, including 50p100K,^44^ ^,^ ^48^ ≥100 viral reads matched at species level^47^ and VirMAP aggregate bit score^42^; several studies did not report thresholds. Thresholds maintain sensitivity while minimising false positives and reducing low‐level cross‐sample contamination risk,^32^ but variability limits comparability, potentially introducing errors into meta‐analysis and its interpretation. Study method heterogeneity was also present, including mixed use of culture, PCR and various mNGS platforms. For example, TEDDY’s use of culture to amplify low‐abundance viruses impeded comparative analysis of viral load and increased bias to detecting certain viruses above threshold.

Despite advances in metagenomics, virome analysis remains challenging, with rapid viral evolution complicating sequencing^77^ and impeding novel virus mapping by bioinformatic databases.^78^ ^,^ ^79^ Indeed in most viral mNGS datasets, more than 50% of sequences exhibit no detectable sequence similarity to known reference sequences, contributing to the viral ‘dark matter'.^80^ It is plausible that these may include highly divergent or completely novel viruses that have yet to be discovered.^81^ Small genomes and low abundance of vertebrate‐infecting viruses in human samples hampers detection, with high background interference from other genetic material.^82^ Thus, effective viral enrichment is necessary, such as enzymatic digestion of non‐viral nucleic acids or size exclusion of non‐viral components via filtration.^31^ Three studies employed VirCapSeq‐VERT and demonstrated enhanced sensitivity for identifying a broader range of vertebrate‐infecting viruses.^43^ ^,^ ^47^ VirCapSeq‐VERT uses approximately two million probes targeting genomes of all known vertebrate‐infecting viruses, increasing the sensitivity of viral sequence detection by up to 10,000‐fold compared to standard mNGS.^32^ Wider application of VirCapSeq‐VERT or other similar pan‐viral enrichment sequencing platforms will significantly enhance the reproducibility and robustness of future virome studies.

Positivity for viral nucleic acid is a marker of infection, not proof, as viruses may pass through the gut without productive infection, as with plant viruses and diet‐derived viruses.^82^, ^83^, ^84^ Similarly, viral shedding in stool/plasma cannot directly evidence pancreatic infection.^85^ Additionally, periodic sample collection precludes determination of first virus exposure, differentiation between persistence or re‐infection over time and definition of precise temporal associations between infection and IA/T1D onset. However, more frequent sampling may not be sustainable over long follow‐up in prospective cohort studies and may still miss some acute infections with a very narrow window for detection.

Studies sampled various body sites through stool, plasma and PBMCs. Direct comparison of across sites is difficult, requiring careful consideration of where viruses replicate. For example, EVs and mastadenoviruses preferentially infect and replicate at mucosal surfaces,^59^ ^,^ ^86^ ^,^ ^87^ and gut viral shedding in stool persists longer, resulting in higher positivity compared to short viraemic periods in plasma/PBMCs.

Finally, this review did not examine bacteriophage to limit scope and adopt the precedent in other studies of focusing on only one class of virus to maximise detection sensitivity.

Overall quality of included studies was high (NOS scores ≥8). All studies except one matched for ≥3 factors. Meta‐analysis demonstrated little significant heterogeneity; however, results must be interpreted cautiously given *χ* ^2^ and *I* ^2^ limitations in detecting true heterogeneity. Studies were small: six had <50 participants, potentially causing small study effects. However, longitudinal sampling increased statistical power for detecting differences in the virome between cases and controls. Thus, we demonstrated the importance of comparing both number of virus‐positive individuals and samples. One study^42^ was significantly larger, with high weighting in meta‐analysis, potentially skewing our results. Influence analyses, removing the smallest and largest outlier studies in turn,^88^ demonstrated insufficient studies of TEDDY scale. The ongoing ENDIA study^89^ will contribute significantly as a large, nationwide observational, prospective cohort of 1,500 children followed from pregnancy through early life.

### 4.2 Future research

Despite limitations of targeted viral detection in IA/T1D pathogenesis studies, there remains a paucity of large, unbiased virome studies. Our findings must be validated in future studies that (1) include >200 participants and frequent longitudinal sampling preceding IA/T1D onset to improve statistical power and counter small‐study effects; (2) include a wider range of HLA genotypes to consider viral associations with IA/T1D in the context of genetic risk; (3) incorporate multicentre data to reduce geographical bias; (4) employ sensitive enrichment and comprehensive sequencing platforms; (5) integrate differential abundance analysis of viral load; and (6) sample various body sites to characterise viral strains and account for niche variations in viral abundance. Future studies should also include virome analysis during pregnancy to explore the role of antenatal and congenital infections in offspring IA/T1D development.^12^

## ACKNOWLEDGEMENTS

This work was supported by the National Health and Medical Research Council Practitioner fellowship APP1136735 (to Maria E. Craig), the Juvenile Diabetes Research Foundation International Postdoctoral Fellowship 3‐PDF‐2020‐940‐A‐N and the Australian Diabetes Society Lindsey Baudinet Award (to Ki Wook Kim). The authors would like to thank Professors Ondrej Cinek and Kendra Vehik for providing supplementary raw data for inclusion in this systematic review.

## CONFLICT OF INTEREST

The author declares that there is no conflict of interests.

## AUTHOR CONTRIBUTIONS

Maria E. Craig and Ki Wook Kim designed the study and led the study group. Clare L. Faulkner performed the literature search, extracted, analysed and interpreted the data and wrote the manuscript. Yi Xuan Luo independently confirmed the database search and study quality. Ki Wook Kim acted as consultant in instances of uncertainty. Yi Xuan Luo, Sonia Isaacs, William D. Rawlinson, Maria E. Craig and Ki Wook Kim helped interpret the data. All authors were involved in the critical revision of this manuscript. Ki Wook Kim is the guarantor of this study.

## Supporting information

Supplementary Material 1

## References

[1] International Diabetes Foundation . IDF Diabetes Atlas. 9th ed. Brussels, Belgium: International Diabetes Foundation; 2019.

[2] Insel RA , Dunne JL , Atkinson MA , et al. Staging presymptomatic type 1 diabetes: a scientific statement of JDRF, the Endocrine Society, and the American Diabetes Association. Diabetes Care. 2015;38(10):1964‐1974. 10.2337/dc15-1419. 26404926 PMC5321245

[3] Ziegler A‐G , Kick K , Bonifacio E , et al. Yield of a public health screening of children for islet autoantibodies in Bavaria, Germany. J Am Med Assoc. 2020;323(4):339‐351. 10.1001/jama.2019.21565. PMC6990943 31990315

[4] Pociot F , Lernmark Å . Genetic risk factors for type 1 diabetes. Lancet. 2016;387(10035):2331‐2339. 10.1016/s0140-6736(16)30582-7. 27302272

[5] Hippich M , Beyerlein A , Hagopian WA , et al. Genetic contribution to the divergence in type 1 diabetes risk between children from the general population and children from affected families. Diabetes. 2019;68(4):847‐857. 10.2337/db18-0882. 30655385 PMC6425872

[6] Craig ME , Kim KW , Isaacs SR , et al. Early‐life factors contributing to type 1 diabetes. Diabetologia. 2019;62(10):1823‐1834. 10.1007/s00125-019-4942-x. 31451871

[7] Krischer JP , Lynch KF , Lernmark Å , et al. Genetic and environmental interactions modify the risk of diabetes‐related autoimmunity by 6 years of age: the TEDDY Study. Diabetes Care. 2017;40(9):1194‐1202. 10.2337/dc17-0238. 28646072 PMC5566280

[8] Haynes A , Bulsara MK , Bergman P , et al. Incidence of type 1 diabetes in 0 to 14 year olds in Australia from 2002 to 2017. Pediatr Diabetes. 2020;21:707‐712. 10.1111/pedi.13025. 32304132

[9] Craig ME , Nair S , Stein H , Rawlinson WD . Viruses and type 1 diabetes: a new look at an old story. Pediatr Diabetes. 2013;14(3):149‐158. 10.1111/pedi.12033. 23517503

[10] Patterson C , Guariguata L , Dahlquist G , Soltész G , Ogle G , Silink M . Diabetes in the young—a global view and worldwide estimates of numbers of children with type 1 diabetes. Diabetes Res Clin Pract. 2014;103(2):161‐175. 10.1016/j.diabres.2013.11.005. 24331235

[11] Beyerlein A , Donnachie E , Jergens S , Ziegler A‐G . Infections in early life and development of type 1 diabetes. J Am Med Assoc. 2016;315(17):1899‐1901. 10.1001/jama.2016.2181. 27139064

[12] Allen DW , Kim KW , Rawlinson WD , Craig ME . Maternal virus infections in pregnancy and type 1 diabetes in their offspring: systematic review and meta‐analysis of observational studies. Rev Med Virol. 2018;28(3):e1974. 10.1002/rmv.1974. 29569297

[13] Sadeharju K , Hamalainen AM , Knip M , et al. Enterovirus infections as a risk factor for type I diabetes: virus analyses in a dietary intervention trial. Clin Exp Immunol. 2003;132(2):271‐277. 10.1046/j.1365-2249.2003.02147.x. 12699416 PMC1808709

[14] Nairn C , Galbraith DN , Taylor KW , Clements GB . Enterovirus variants in the serum of children at the onset of type 1 diabetes mellitus. Diabet Med. 1999;16(6):509‐513. 10.1046/j.1464-5491.1999.00098.x. 10391400

[15] Honkanen H , Oikarinen S , Nurminen N , et al. Detection of enteroviruses in stools precedes islet autoimmunity by several months: possible evidence for slowly operating mechanisms in virus‐induced autoimmunity. Diabetologia. 2017;60(3):424‐431. 10.1007/s00125-016-4177-z. 28070615

[16] Oikarinen S , Martiskainen M , Tauriainen S , et al. Enterovirus RNA in blood is linked to the development of type 1 diabetes. Diabetes. 2011;60(1):276‐279. 10.2337/db10-0186. 20943747 PMC3012181

[17] Cinek O , Stene LC , Kramná L , et al. Enterovirus RNA in longitudinal blood samples and risk of islet autoimmunity in children with a high genetic risk of type 1 diabetes: the MIDIA study. Diabetologia. 2014;57(10):2193‐2200. 10.1007/s00125-014-3327-4. 25047648

[18] Yeung WCG , Rawlinson WD , Craig ME . Enterovirus infection and type 1 diabetes mellitus: systematic review and meta‐analysis of observational molecular studies. Br Med J. 2011;342:d35. 10.1136/bmj.d35. 21292721 PMC3033438

[19] Richardson SJ , Willcox A , Bone AJ , Foulis AK , Morgan NG . The prevalence of enteroviral capsid protein VP1 immunostaining in pancreatic islets in human type 1 diabetes. Diabetologia. 2009;52(6):1143‐1151. 10.1007/s00125-009-1276-0. 19266182

[20] Krogvold L , Edwin B , Buanes T , et al. Detection of a low‐grade enteroviral infection in the islets of Langerhans of living patients newly diagnosed with type 1 diabetes. Diabetes. 2015;64(5):1682‐1687. 10.2337/db14-1370. 25422108

[21] Tapia G , Cinek O , Rasmussen T , et al. Human enterovirus RNA in monthly fecal samples and islet autoimmunity in Norwegian children with high genetic risk for type 1 diabetes: the MIDIA study. Diabetes Care. 2011;34(1):151‐155. 10.2337/dc10-1413. 20929993 PMC3005474

[22] Rodriguez‐Calvo T . Enterovirus infection and type 1 diabetes: unraveling the crime scene. Clin Exp Immunol. 2019;195(1):15‐24. 10.1111/cei.13223. 30307605 PMC6300647

[23] Green J , Casabonne D , Newton R . Coxsackie B virus serology and type 1 diabetes mellitus: a systematic review of published case‐control studies. Diabet Med. 2004;21(6):507‐514. 10.1111/j.1464-5491.2004.01182.x. 15154932

[24] Hyöty H , Leinikki P , Reunanen A , et al. Mumps infections in the etiology of type 1 (insulin‐dependent) diabetes. Diabetes Res. 1988;9(3):111‐116. 3243043

[25] Ekman I , Vuorinen T , Knip M , et al. Early childhood CMV infection may decelerate the progression to clinical type 1 diabetes. Pediatr Diabetes. 2019;20(1):73‐77. 10.1111/pedi.12788. 30338642

[26] Honeyman MC , Coulson BS , Stone NL , et al. Association between rotavirus infection and pancreatic islet autoimmunity in children at risk of developing type 1 diabetes. Diabetes. 2000;49(8):1319‐1324. 10.2337/diabetes.49.8.1319. 10923632

[27] Tauriainen S , Martiskainen M , Oikarinen S , et al. Human parechovirus 1 infections in young children ‐ no association with type 1 diabetes. J Med Virol. 2007;79(4):457‐462. 10.1002/jmv.20831. 17311340

[28] Kolehmainen P , Koskiniemi M , Oikarinen S , et al. Human parechovirus and the risk of type 1 diabetes. J Med Virol. 2013;85(9):1619‐1623. 10.1002/jmv.23659. 23852688

[29] Bian X , Wallstrom G , Davis A , et al. Immunoproteomic profiling of antiviral antibodies in new‐onset type 1 diabetes using protein arrays. Diabetes. 2016;65(1):285‐296. 10.2337/db15-0179. 26450993 PMC4686945

[30] Munakata Y , Kodera T , Saito T , Sasaki T . Rheumatoid arthritis, type 1 diabetes, and Graves' disease after acute parvovirus B19 infection. Lancet. 2005;366(9487):P780. 10.1016/S0140-6736(05)67184-X. 16125597

[31] Kumar A , Murthy S , Kapoor A . Evolution of selective‐sequencing approaches for virus discovery and virome analysis. Virus Res 2017;239:172‐179. 10.1016/j.virusres.2017.06.005. 28583442 PMC5819613

[32] Briese T , Kapoor A , Mishra N , et al. Virome capture sequencing enables sensitive viral diagnosis and comprehensive virome analysis. mBio. 2015;6(5)‐e01491‐15. 10.1128/mbio.01491-15. PMC4611031 26396248

[33] American Diabetes Association . Classification and diagnosis of diabetes: standards of medical care in diabetes—2019. Diabetes Care. 2019;42(suppl 1):S13‐S28. 10.2337/dc19-s002. 30559228

[34] Merlin T , Weston A , Tooher R . Extending an evidence hierarchy to include topics other than treatment: revising the Australian 'levels of evidence. BMC Med Res Methodol. 2009;9(1):34. 10.1186/1471-2288-9-34. 19519887 PMC2700132

[35] Wells GA , Shea B , O'Connell D , et al. The Newcastle‐Ottawa Scale (NOS) for assessing the quality of nonrandomised studies in meta‐analyses; 2000. http://www.ohri.ca/programs/clinical_epidemiology/oxford.asp. Accessed June 1, 2020.

[36] Review Manager (Revman), Version 5.4 [computer program]. Copenhagen, Denmark: The Cochrane Collaboration; 2014.

[37] Cinek O , Kramná L , Lin J , et al. The microbiome in children with islet autoimmunity: bacteriome profiling and virome sequencing in stool samples from Finnish DIPP study. Pediatr Diabetes. 2016;17(suppl 24):S24. 10.1111/pedi.12450. 27860030

[38] Cinek O , Kramná L , Lin J , et al. Imbalance of bacteriome profiles in children with islet autoimmunity: mass sequencing of 16S DNA and viromes in stool samples from Finnish DIPP study. Diabetologia. 2016;59(suppl 1):S185‐S186. 10.1007/s00125-016-4046-9.

[39] Kramná L , Holkova K , Oikarinen S , et al. Mass sequencing of the faecal virome: a study in children with early onset of islet autoimmunity. Diabetologia. 2014;57(suppl 1):S78. 10.1007/s00125-014-3355-0.

[40] Simonen‐Tikka ML , Pflueger M , Klemola P , et al. Human enterovirus infections in children at increased risk for type 1 diabetes: the Babydiet study. Diabetologia. 2011;54(12):2995‐3002. 10.1007/s00125-011-2305-3. 21932150

[41] Cinek O , Kramná L , Mazankova K , et al. Gut bacteriome and virome at the onset of type 1 diabetes: a study from four geographically distant African and Asian countries. Pediatr Diabetes. 2017;18(suppl 25):S44. 10.1111/pedi.12587. 33786936

[42] Vehik K , Lynch KF , Wong MC , et al. Prospective virome analyses in young children at increased genetic risk for type 1 diabetes. Nat Med. 2019;25(12):1865‐1872. 10.1038/s41591-019-0667-0. 31792456 PMC6898786

[43] Hippich M , Oleynik A , Jain K , et al. Searching peripheral blood mononuclear cells of children with viral respiratory tract infections preceding islet autoimmunity for viruses by high‐throughput sequencing. Acta Diabetol. 2018;55(8):881‐884. 10.1007/s00592-018-1138-7. 29687279

[44] Kramná L , Kolarova K , Oikarinen S , et al. Gut virome sequencing in children with early islet autoimmunity. Diabetes Care. 2015;38(5):930‐933. 10.2337/dc14-2490. 25678103

[45] Lee HS , Briese T , Winkler C , et al. Next‐generation sequencing for viruses in children with rapid‐onset type 1 diabetes. Diabetologia. 2013;56(8):1705‐1711. 10.1007/s00125-013-2924-y. 23657799 PMC4019381

[46] Zhao G , Vatanen T , Droit L , et al. Intestinal virome changes precede autoimmunity in type I diabetes‐susceptible children. Proc Natl Acad Sci USA. 2017;114(30):E6166‐E6175. 10.1073/pnas.1706359114. 28696303 PMC5544325

[47] Kim KW , Horton JL , Pang CNI , et al. Higher abundance of enterovirus A species in the gut of children with islet autoimmunity. Sci Rep. 2019;9(1):1749. 10.1038/s41598-018-38368-8. 30741981 PMC6370883

[48] Cinek O , Kramná L , Lin J , et al. Imbalance of bacteriome profiles within the Finnish Diabetes Prediction and Prevention study: parallel use of 16S profiling and virome sequencing in stool samples from children with islet autoimmunity and matched controls. Pediatr Diabetes. 2017;18(7):588‐598. 10.1111/pedi.12468. 27860030

[49] Salminen K , Sadeharju K , Lönnrot M , et al. Enterovirus infections are associated with the induction of β‐cell autoimmunity in a prospective birth cohort study. J Med Virol. 2003;69(1):91‐98. 10.1002/jmv.10260. 12436483

[50] Sarmiento L , Cabrera‐Rode E , Lekuleni L , et al. Occurrence of enterovirus RNA in serum of children with newly diagnosed type 1 diabetes and islet cell autoantibody‐positive subjects in a population with a low incidence of type 1 diabetes. Autoimmunity. 2007;40(7):540‐545. 10.1080/08916930701523429. 17966045

[51] Laitinen OH , Honkanen H , Pakkanen O , et al. Coxsackievirus B1 is associated with induction of β‐cell autoimmunity that portends type 1 diabetes. Diabetes. 2014;63(2):446‐455. 10.2337/db13-0619. 23974921

[52] Schulte BM , Bakkers J , Lanke KHW , et al. Detection of enterovirus RNA in peripheral blood mononuclear cells of type 1 diabetic patients beyond the stage of acute infection. Viral Immunol 2010;23(1):99‐104. 10.1089/vim.2009.0072. 20121407

[53] Ylipaasto P , Klingel K , Lindberg AM , et al. Enterovirus infection in human pancreatic islet cells, islet tropism in vivo and receptor involvement in cultured islet beta cells. Diabetologia. 2004;47(2):225‐239. 10.1007/s00125-003-1297-z. 14727023

[54] Richardson SJ , Leete P , Bone AJ , Foulis AK , Morgan NG . Expression of the enteroviral capsid protein VP1 in the islet cells of patients with type 1 diabetes is associated with induction of protein kinase R and downregulation of Mcl‐1. Diabetologia. 2013;56(1):185‐193. 10.1007/s00125-012-2745-4. 23064357

[55] Richardson SJ , Morgan NG . Enteroviral infections in the pathogenesis of type 1 diabetes: new insights for therapeutic intervention. Curr Opin Pharmacol. 2018;43:11‐19. 10.1016/j.coph.2018.07.006. 30064099 PMC6294842

[56] Genoni A , Canducci F , Rossi A , et al. Revealing enterovirus infection in chronic human disorders: an integrated diagnostic approach. Sci Rep. 2017;7(1):5013. 10.1038/s41598-017-04993-y. 28694527 PMC5504018

[57] Dunne JL , Richardson SJ , Atkinson MA , et al. Rationale for enteroviral vaccination and antiviral therapies in human type 1 diabetes. Diabetologia. 2019;62(5):744‐753. 10.1007/s00125-019-4811-7. 30675626 PMC6450860

[58] Petzold A , Solimena M , Knoch K‐P . Mechanisms of beta cell dysfunction associated with viral infection. Curr Diab Rep. 2015;15(10):73‐82. 10.1007/s11892-015-0654-x. 26280364 PMC4539350

[59] Oikarinen M , Tauriainen S , Oikarinen S , et al. Type 1 diabetes is associated with enterovirus infection in gut mucosa. Diabetes. 2012;61(3):687‐691. 10.2337/db11-1157. 22315304 PMC3282798

[60] Op de Beeck A , Eizirik DL . Viral infections in type 1 diabetes mellitus—why the β cells? Nat Rev Endocrinol. 2016;12(5):263‐273. 10.1038/nrendo.2016.30. 27020257 PMC5348720

[61] Kanno T , Kim K , Kono K , Drescher KM , Chapman NM , Tracy S . Group B coxsackievirus diabetogenic phenotype correlates with replication efficiency. J Virol. 2006;80(11):5637‐5643. 10.1128/JVI.02361-05. 16699045 PMC1472143

[62] Kracht MJ , van Lummel M , Nikolic T , et al. Autoimmunity against a defective ribosomal insulin gene product in type 1 diabetes. Nat Med. 2017;23(4):501‐507. 10.1038/nm.4289. 28263308

[63] Filippi CM , Von Herrath MG . Viral trigger for type 1 diabetes: pros and cons. Diabetes. 2008;57(11):2863‐2871. 10.2337/db07-1023. 18971433 PMC2570378

[64] Poole C , Greenland S . Random‐effects meta‐analyses are not always conservative. Am J Epidemiol. 1999;150(5):469‐475. 10.1093/oxfordjournals.aje.a010035. 10472946

[65] Higgins J , Thomas J , Chandler J , et al. Cochrane Handbook for Systematic Reviews of Interventions (Version 6.1). Cochrane; 2020. www.training.cochrane.org/handbook. Accessed November 25, 2020.

[66] Endesfelder D , Zu Castell W , Bonifacio E , et al. Time‐resolved autoantibody profiling facilitates stratification of preclinical type 1 diabetes in children. Diabetes. 2019;68(1):119‐130. 10.2337/db18-0594. 30305370 PMC6302536

[67] Steck AK , Vehik K , Bonifacio E , et al. Predictors of progression from the appearance of islet autoantibodies to early childhood diabetes: the Environmental Determinants of Diabetes in the Young (TEDDY). Diabetes Care. 2015;38(5):808‐813. 10.2337/dc14-2426. 25665818 PMC4407751

[68] Thomson G , Valdes AM , Noble JA , et al. Relative predispositional effects of HLA class II DRB1‐DQB1 haplotypes and genotypes on type 1 diabetes: a meta‐analysis. Tissue Antigens. 2007;70(2):110‐127. 10.1111/j.1399-0039.2007.00867.x. 17610416

[69] Craig ME , Howard NJ , Silink M , Rawlinson WD . Reduced frequency of HLA DRB1*03‐DQB1*02 in children with type 1 diabetes associated with enterovirus RNA. J Infect Dis. 2003;187(10):1562‐1570. 10.1086/374742. 12721936

[70] Witsø E , Cinek O , Tapia G , Rasmussen T , Stene LC , Rønningen KS . HLA‐DRB1‐DQA1‐DQB1 genotype and frequency of enterovirus in longitudinal monthly fecal samples from healthy infants. Viral Immunol 2012;25(3):187‐192. 10.1089/vim.2012.0001. 22691100

[71] Dahlquist G . Can we slow the rising incidence of childhood‐onset autoimmune diabetes? The overload hypothesis. Diabetologia. 2006;49(1):20‐24. 10.1007/s00125-005-0076-4. 16362279

[72] Lamb MM , Miller M , Seifert JA , et al. The effect of childhood cow's milk intake and HLA‐DR genotype on risk of islet autoimmunity and type 1 diabetes: the Diabetes Autoimmunity Study in the Young. Pediatr Diabetes. 2015;16(1):31‐38. 10.1111/pedi.12115. 24444005 PMC4104257

[73] Dong JY , Zhang WG , Chen JJ , Zhang ZL , Han SF , Qin LQ . Vitamin D intake and risk of type 1 diabetes: a meta‐analysis of observational studies. Nutrients. 2013;5(9):3551‐3562. 10.3390/nu5093551. 24036529 PMC3798920

[74] Norris JM , Yin X , Lamb MM , et al. Omega‐3 polyunsaturated fatty acid intake and islet autoimmunity in children at increased risk for type 1 diabetes. J Am Med Assoc. 2007;298(12):1420‐1428. 10.1001/jama.298.12.1420. 17895458

[75] Bonifacio E , Warncke K , Winkler C , Wallner M , Ziegler AG . Cesarean section and interferon‐induced helicase gene polymorphisms combine to increase childhood type 1 diabetes risk. Diabetes. 2011;60(12):3300‐3306. 10.2337/db11-0729. 22110093 PMC3219940

[76] Frederiksen B , Kroehl M , Lamb MM , et al. Infant exposures and development of type 1 diabetes mellitus: the Diabetes Autoimmunity Study in the Young (DAISY). JAMA Pediatr 2013;167(9):808‐815. 10.1001/jamapediatrics.2013.317. 23836309 PMC4038357

[77] Minot S , Bryson A , Chehoud C , Wu GD , Lewis JD , Bushman FD . Rapid evolution of the human gut virome. Proc Natl Acad Sci USA. 2013;110(30):12450‐12455. 10.1073/pnas.1300833110. 23836644 PMC3725073

[78] Shkoporov AN , Clooney AG , Sutton TDS , et al. The human gut virome is highly diverse, stable, and individual specific. Cell Host Microb. 2019;26(4):527‐541. 10.1016/j.chom.2019.09.009. 31600503

[79] Garmaeva S , Sinha T , Kurilshikov A , Fu J , Wijmenga C , Zhernakova A . Studying the gut virome in the metagenomic era: challenges and perspectives. BMC Biol. 2019;17(1):84‐98. 10.1186/s12915-019-0704-y. 31660953 PMC6819614

[80] Roux S , Hallam SJ , Woyke T , Sullivan MB . Viral dark matter and virus–host interactions resolved from publicly available microbial genomes. eLife. 2015;4:e08490. 10.7554/elife.08490. PMC4533152 26200428

[81] Wang D . 5 challenges in understanding the role of the virome in health and disease. PLoS Pathog. 2020;16(3):e1008318. 10.1371/journal.ppat.1008318. 32214384 PMC7098563

[82] Kim KW , Allen DW , Briese T , et al. Higher frequency of vertebrate‐infecting viruses in the gut of infants born to mothers with type 1 diabetes. Pediatr Diabetes. 2020;21(2):271‐279. 10.1111/pedi.12952. 31800147

[83] Zhang T , Breitbart M , Lee WH , et al. RNA viral community in human feces: prevalence of plant pathogenic viruses. PLoS Biol. 2005;4(1):108‐118. 10.1371/journal.pbio.0040003. PMC1310650 16336043

[84] Kim KW , Allen DW , Briese T , et al. Distinct gut virome profile of pregnant women with type 1 diabetes in the ENDIA study. Open Forum Infect Dis. 2019;6(2):ofz025. 10.1093/ofid/ofz025. PMC6386807 30815502

[85] Carding SR , Davis N , Hoyles L . Review article: the human intestinal virome in health and disease. Aliment Pharmacol Ther. 2017;46(9):800‐815. 10.1111/apt.14280. 28869283 PMC5656937

[86] Rodriguez‐Calvo T , Sabouri S , Anquetil F , Von Herrath MG . The viral paradigm in type 1 diabetes: who are the main suspects? Autoimmun Rev. 2016;15(10):964‐969. 10.1016/j.autrev.2016.07.019. 27491567

[87] Chung PW , Huang YC , Chang LY , Lin TY , Ning HC . Duration of enterovirus shedding in stool. J Microbiol Immunol Infect. 2001;34(3):167‐170. 11605806

[88] Viechtbauer W , Cheung MWL . Outlier and influence diagnostics for meta‐analysis. Res Synth Methods. 2010;1(2):112‐125. 10.1002/jrsm.11. 26061377

[89] Penno MA , Couper JJ , Craig ME , et al. Environmental determinants of islet autoimmunity (ENDIA): a pregnancy to early life cohort study in children at‐risk of type 1 diabetes. BMC Pediatr. 2013;13(1):124. 10.1186/1471-2431-13-124. 23941366 PMC3751791

