# Supplementary material for: The virome in early life and childhood and development of islet autoimmunity and type 1 diabetes: A systematic review and meta‐analysis of observational studies
Source: Rev Med Virol. 2020 Dec 30;31(5):e2209. doi: 10.1002/rmv.2209 (PMC8518965; doi:10.1002/rmv.2209)
Supplement: Supplementary file 1 — Supplementary Material 1 [file RMV-31-e2209-s001.docx]

**Supplementary Material**

**Appendix A – Search Strategy**

The database searches were performed on 1^st^ June 2020.

**Medline**

| **Step** | **Search** |
| --- | --- |
| 1 | *Diabetes Mellitus, Type 1/ or (Type 1 diabetes or Type one diabetes or Type I diabetes).mp. or exp insulin dependent diabetes mellitus/ or insulin dependent diabetes.mp. or juvenile diabetes.mp. |
| 2 | Autoimmunity/ or Autoantibodies/ or islet autoimmun*.mp. or Islets of Langerhans/ or (insulin autoantibody* or insulin antibod*).mp. |
| 3 | Child/ OR Child, Preschool/ |
| 4 | Infant/ OR Infant, Newborn/ |
| 5 | Viruses/ or dna viruses/ or rna viruses/ |
| 6 | virome*.mp. |
| 7 | Viral Load/ |
| 8 | Virus Diseases/ |
| 9 | virus*.mp. |
| 10 | high-throughput nucleotide sequencing/ or metagenomics/ or virome capture.mp. |
| 11 | serology.mp. or Serology/ |
| 12 | Serologic Tests/ |
| 13 | 1 or 2 |
| 14 | 3 or 4 |
| 15 | 5 or 6 or 7 or 8 or 9 or 10 |
| 16 | 11 or 12 |
| 17 | 13 and 14 and 15 |
| 18 | 16 not 17 |
| 19 | 17 not 18 |
| 20 | Limit 19 to “review” |
| 21 | 19 not 20 |
| 22 | Limit 21 to humans |
| 23 | Limit 22 to yr=”2000-Current” |

**Embase**

| **Step** | **Search** |
| --- | --- |
| 1 | Exp Insulin dependent diabetes mellitus/ or insulin dependent diabetes.mp. or (Type 1 diabetes or Type one diabetes or Type I diabetes).mp. or juvenile diabetes.mp. or *insulin dependent diabetes mellitus/ |
| 2 | Autoimmunity/ or autoantibody/ or islet autoimmunity.mp. or pancreas islet/ or (insulin autoantibody* or insulin antibod*).mp. |
| 3 | Child/ or school child/ or preschool child/ |
| 4 | Infant/ or Newborn/ |
| 5 | Virus infection/ or DNA virus infection/ or virus load/ or virus/ or RNA virus infection/ or enteric virus/ or DNA virus/ or RNA virus/ or virome*.mp. |
| 6 | Metagenomics/ or Next generation sequencing/ or high throughput sequencing/ or virome capture.mp. |
| 7 | Serology/ or serology.mp. or Serologic Tests/ |
| 8 | 1 or 2 |
| 9 | 3 or 4 |
| 10 | 5 or 6 |
| 11 | 8 and 9 and 10 |
| 12 | 7 and 11 |
| 13 | 11 not 12 |
| 14 | Limit 13 to “review” |
| 15 | 13 not 14 |
| 16 | Limit 15 to human |
| 17 | Limit 16 to yr=”2000-Current” |

**Supplementary Table 1**

**Supplementary Table 1** Meta-analyses of specified individual viruses and IA development, including for number of individuals positive and number of samples positive for the virus

| **Virus*** | **Type of studies** | **Number of studies** | **Number of individuals positive** | | | **Number of samples positive** | | |
| --- | --- | --- | --- | --- | --- | --- | --- | --- |
|  |  |  | **OR (95% CI)** | **P value** | **Heterogeneity** | **OR (95% CI)** | **P value** | **Heterogeneity** |
| Enterovirus | Any sample | 6 | 1.13 (0.86-1.48) | 0.37 | X^2^ = 0.51; P = 0.77; I^2^ = 0% | 1.13 (1.00-1.28) | 0.05 | X^2^ = 0.52; P = 0.76; I^2^ = 0% |
| Enterovirus | Stool | 4 | 1.15 (0.87-1.51) | 0.32 | X^2^ = 0.48; P = 0.69; I^2^ = 0% | 1.14 (1.00-1.29) | 0.05 | X^2^ = 0.50; P = 0.68; I^2^ = 0% |
| Enterovirus | Plasma | 2 | 0.80 (0.23-2.77) | 0.72 | X^2^ = 0.81; P = 0.37; I^2^ = 0% | 0.80 (0.24-2.73) | 0.73 | X^2^ = 0.78; P = 0.38; I^2^ = 0% |
| Enterovirus A | Stool | 2 | 1.12 (0.84-1.50) | 0.42 | X^2^ = 0.13; P = 0.72; I^2^ = 0% | 1.61 (0.43-5.94) | 0.48 | X^2^ = 2.81; P = 0.09; I^2^ = 64% |
| Enterovirus B | Stool | 2 | 0.99 (0.74-1.32) | 0.94 | X^2^ = 0.24; P = 0.62; I^2^ = 0% | 1.20 (1.01-1.42) | 0.04 | X^2^ = 0.03; P = 0.86; I^2^ = 0% |
| Parechovirus | Stool | 4 | 0.83 (0.63-1.10) | 0.20 | X^2^ = 0.46; P = 0.71; I^2^ = 0% | 0.66 (0.32-1.35) | 0.25 | X^2^ = 3.88; P = 0.009; I^2^ = 74% |
| Rotavirus | Any sample | 3 | 0.48 (0.12-1.97) | 0.31 | X^2^ = 0.79; P = 0.45; I^2^ = 0% | N/A | N/A | N/A |
| Rotavirus | Stool | 2 | 0.31 (0.07-1.50) | 0.15 | X^2^ = 0.00; P = 0.99; I^2^ = 0% | 0.75 (0.29-1.94) | 0.55 | X^2^ = 0.29; P = 0.59; I^2^ = 0% |
| Bocaparvovirus | Any sample | 5 | 0.95 (0.72-1.25) | 0.70 | X^2^ = 0.53; P = 0.71; I^2^ = 0% | 0.94 (0.81-1.08) | 0.37 | X^2^ = 0.39; P = 0.82; I^2^ = 0% |
| Bocaparvovirus | Stool | 4 | 0.93 (0.71-1.23) | 0.63 | X^2^ = 0.31; P = 0.82; I^2^ = 0% | 0.93 (0.81-1.08) | 0.34 | X^2^ = 0.11; P = 0.95; I^2^ = 0% |
| Anelloviridae^ | Any sample | 5 | 1.02 (0.72-1.44) | 0.91 | X^2^ = 0.69; P = 0.60; I^2^ = 0% | 1.06 (0.84-1.34) | 0.61 | X^2^ = 0.87; P = 0.48; I^2^ = 0% |
| Anelloviridae^ | Stool | 4 | 1.08 (0.74-1.57) | 0.68 | X^2^ = 0.69; P = 0.56; I^2^ = 0% | 1.09 (0.86-1.39) | 0.48 | X^2^ = 0.94; P = 0.42; I^2^ = 0% |
| Sapovirus | Stool | 3 | 1.09 (0.81-1.47) | 0.55 | X^2^ = 0.21; P = 0.81; I^2^ = 0% | 1.03 (0.86-1.25) | 0.74 | X^2^ = 0.22; P = 0.80; I^2^ = 0% |
| Norovirus | Stool | 2 | 0.47 (0.06-3.79) | 0.48 | X^2^ = 2.34; P = 0.13; I^2^ = 57% | 0.51 (0.06-4.12) | 0.53 | X^2^ = 2.39; P = 0.12; I^2^ = 58% |
| Cardiovirus | Any sample | 3 | 0.76 (0.25-2.32) | 0.63 | X^2^ = 1.29; P = 0.28; I^2^ = 22% | 0.79 (0.26-2.39) | 0.68 | X^2^ = 1.31; P = 0.27; I^2^ = 23% |
| Cardiovirus | Stool | 2 | 0.62 (0.09-4.31) | 0.63 | X^2^ = 2.04; P = 0.15; I^2^ = 51% | 0.66 (0.10-4.35) | 0.66 | X^2^ = 2.04; P = 0.15; I^2^ = 51% |
| Circovirus | Stool | 2 | 0.24 (0.02-2.74) | 0.25 | X^2^ = 1.94; P = 0.16; I^2^ = 48% | 0.16 (0.01-4.66) | 0.28 | X^2^ = 4.06; P = 0.04; I^2^ = 75% |
| Mamastrovirus | Stool | 2 | 1.15 (0.53-2.50) | 0.73 | X^2^ = 1.11; P = 0.29; I^2^ = 10% | 1.11 (0.71-1.74) | 0.64 | X^2^ = 1.04; P = 0.31; I^2^ = 4% |
| Kobuvirus | Stool | 2 | 1.05 (0.23-4.77) | 0.95 | X^2^ = 0.66; P = 0.42; I^2^ = 0% | 1.81 (0.22-15.17) | 0.58 | X^2^ = 1.97; P = 0.16; I^2^ = 49% |
| Mastadenovirus | Any sample | 3 | 1.05 (0.76-1.45) | 0.76 | X^2^ = 0.85; P = 0.43; I^2^ = 0% | 1.06 (0.96-1.16) | 0.26 | X^2^ = 0.82; P = 0.44; I^2^ = 0% |
| Mastadenovirus | Stool | 2 | 1.03 (0.74-1.42) | 0.87 | X^2^ = 0.00; P = 0.97; I^2^ = 0% | 1.05 (0.96-1.16) | 0.28 | X^2^ = 0.00; P = 0.94; I^2^ = 0% |
| Picobirnavirus | Stool | 2 | 0.74 (0.11-5.22) | 0.77 | X^2^ = 1.43; P = 0.23; I^2^ = 30% | 0.45 (0.05-4.18) | 0.48 | X^2^ = 2.10; P = 0.15; I^2^ = 52% |
| Erythroparvovirus | Plasma | 2 | 1.84 (0.37-9.18) | 0.46 | X^2^ = 0.15; P = 0.70; I^2^ = 0% | N/A | N/A | N/A |
| Roseolovirus | Plasma | 2 | 0.61 (0.07-5.16) | 0.65 | X^2^ = 0.28; P = 0.59; I^2^ = 0% | N/A | N/A | N/A |

*Most viruses are reported at genus level, except for enterovirus A and enterovirus B reported at species level; ^Variable reporting of anelloviruses limited analysis to family-level; N/A = not available

**Supplementary Table 2**

**Supplementary Table 2** Meta-analyses of studies investigating the number of individuals with consecutive shedding of the same viral genus and IA development

| **Virus** | **Type of studies** | **Number of studies** | **Number of individuals with ≥2 consecutive samples positive for the same viral genus** | | |
| --- | --- | --- | --- | --- | --- |
|  |  |  | **OR (95% CI)** | **P value** | **Heterogeneity** |
| Enterovirus | Stool | 3 | 1.55 (1.09-2.20) | 0.01 | X^2^ = 0.10; P = 0.91; I^2^ = 0% |
| Parechovirus | Stool | 2 | 0.89 (0.66-1.20) | 0.45 | X^2^ = 0.53; P = 0.47; I^2^ = 0% |
| Bocaparvovirus | Stool | 2 | 1.18 (0.74-1.88) | 0.48 | X^2^ = 0.02; P = 0.88; I^2^ = 0% |
| Anelloviridae* | Any sample type | 3 | 1.19 (0.51-2.77) | 0.69 | X^2^ = 0.36; P = 0.70; I^2^ = 0% |
| Anelloviridae* | Stool | 2 | 1.21 (0.50-2.93) | 0.67 | X^2^ = 0.71; P = 0.40; I^2^ = 0% |
| Picobirnavirus | Stool | 2 | 0.60 (0.07-5.21) | 0.65 | X^2^ = 0.29; P = 0.59; I^2^ = 0% |

*Variable reporting of anelloviruses limited analysis to family-level

**Supplementary Table 3**

**Supplementary Table 3** Meta-analyses of the viruses detected by the two studies applying the same positivity threshold of 50 viral reads per 100 raw reads and IA development

| **Virus** | **Type of studies** | **Number of studies** | **OR (95% CI)** | **P-value** | **Heterogeneity** |
| --- | --- | --- | --- | --- | --- |
| Enterovirus | Stool | 2 | 1.36 (0.14-12.92) | 0.79 | X^2^ = 1.32; P = 0.25; I^2^ = 24% |
| Parechovirus | Stool | 2 | 1.01 (0.22-4.62) | 0.99 | X^2^ = 0.57; P = 0.45; I^2^ = 0% |
| Anelloviruses | Stool | 2 | 0.65 (0.10-4.26) | 0.66 | X^2^ = 0.15; P = 0.70; I^2^ = 0% |
| Bocaparvovirus | Stool | 2 | 0.41 (0.06-2.95) | 0.37 | X^2^ = 0.04; P = 0.85; I^2^ = 0% |
| Sapovirus | Stool | 2 | 1.00 (0.10-10.04) | 1.00 | X^2^ = 0.95; P = 0.33; I^2^ = 0% |

**Supplementary Table 4**

**Supplementary Table 4** Meta-analyses of specified individual viruses and T1D development, analysed by number of individuals positive

| **Virus** | **Type of studies** | **Number of studies** | **Number of individuals positive** | | |
| --- | --- | --- | --- | --- | --- |
|  |  |  | **OR (95% CI)** | **P-value** | **Heterogeneity** |
| Enterovirus | Stool | 2 | 0.52 (0.12-2.31) | 0.39 | X^2^ = 2.22; P = 0.14; I^2^ = 55% |
| Parechovirus | Stool | 2 | 0.82 (0.47-1.42) | 0.47 | X^2^ = 0.49; P = 0.49; I^2^ = 0% |
| Cardiovirus | Stool | 2 | 0.66 (0.30-1.47) | 0.31 | X^2^ = 0.33; P = 0.57; I^2^ = 0% |
| Norovirus | Stool | 2 | 0.98 (0.58-1.65) | 0.94 | X^2^ = 0.83; P = 0.36; I^2^ = 0% |
| Sapovirus | Stool | 2 | 1.27 (0.75-2.14) | 0.37 | X^2^ = 0.02; P = 0.89; I^2^ = 0% |
| Mastadenovirus | Stool | 2 | 0.91 (0.48-1.75) | 0.79 | X^2^ = 0.11; P = 0.74; I^2^ = 0% |
| Human mastadenovirus A | Stool | 2 | 0.92 (0.51-1.65) | 0.78 | X^2^ = 0.94; P = 0.33; I^2^ = 0% |
| Human mastadenovirus F | Stool | 2 | 0.72 (0.42-1.24) | 0.24 | X^2^ = 0.07; P = 0.79; I^2^ = 0% |
| Bocaparvovirus | Stool | 2 | 1.17 (0.70-1.96) | 0.56 | X^2^ = 0.31; P = 0.58; I^2^ = 0% |
| Mamastrovirus | Stool | 2 | 0.66 (0.37-1.18) | 0.16 | X^2^ = 0.32; P = 0.57; I^2^ = 0% |
| Anelloviridae* | Stool | 2 | 0.83 (0.46-1.52) | 0.55 | X^2^ = 0.52; P = 0.47; I^2^ = 0% |

*Variable reporting of anelloviruses limited analysis to family-level
